# Pathophysiology and epidemiology of virus-induced asthma

**DOI:** 10.3389/fmicb.2014.00562

**Published:** 2014-10-22

**Authors:** Hirokazu Kimura, Akihide Ryo

**Affiliations:** ^1^Infectious Disease Surveillance Center, National Institute of Infectious DiseasesTokyo, Japan; ^2^Department of Molecular Biodefence Research, Yokohama City University Graduate School of MedicineKanagawa, Japan

**Keywords:** virus-induced asthma, epidemiology, pathology, respiratory virus, human immunity

Many respiratory viruses are mainly responsible for common cold, bronchitis, bronchiolitis, and pneumonia. Furthermore, asthma and chronic obstructive pulmonary disease (COPD) are major cause of mortality. The prevalence of asthma in developed countries is approximately 10% in adults and even higher in children (Barnes, 2008). Thus, the medical costs for these diseases are a major burden in many countries. Respiratory virus infections also cause the most of acute exacerbation of asthma (virus-induced asthma) or COPD. Among them, human rhinoviruses (HRV) are detected in the two thirds of the cases with asthma exacerbations in children (Johnston et al., 1995). However, epidemiology and pathophysiology of asthma and COPD is not known. Furthermore, a few effective vaccines have been applied. Therefore, it may be important to better understand pathophysiology of virus-induced asthma or virus-induced COPD exacerbation. Both aspects of the virus agents and host defense systems including acute/chronic inflammation and airway tissue remodeling should be clarified. This e-book aims to review and discuss pathophysiology and epidemiology of virus-induced asthma and COPD focusing on new findings of the host immunity and virology.

This Research Topic contains 7 review articles and 3 original articles regarding pathophysiology of virus-induced asthma. As the first article, Kudo et al. (2013) reviewed pathology of asthma. This article globally covers from molecular histopathology involved in cytokine networks of asthma. The readers may easily understand molecular immunopathology of virus-induced asthma. In the second issue, Okayama (2013) presents cellular and humoral immunity of asthma. Accumulating evidence implicates that the genetic and environmental factors may be associated with virus-induced asthma. This work focuses on the immunological mechanisms that may explain why asthma is associated with RSV-and HRV-infection. As the third review article, Kimura et al. (2013) present the molecular mechanisms between various cytokines and innate immunity of viral respiratory infections including virus-induced asthma. The authors also show the signaling pathways with regard to them. In the 4th review article, Tsukagoshi et al. (2013) discuss the genetic characteristics and molecular evolution of respiratory viruses, and epidemiology of asthma. They also show phylogenetic analysis of the detected viruses in the children with respiratory syncytial virus- (RSV) and/or HRV-associated wheezing and asthma. As the 5th review article, Inoue and Shimojo (2013) present epidemiology and pathophysiology of virus-induced asthma in children. They summarize the previous findings and discuss how clinicians can effectively intervene in these viral infections to prevent the development of asthma. Next, Kurai et al. (2013) and Saraya et al. (2014) present pathophysiology of virus-induced COPD and asthma in adults. They summarize current knowledge concerning exacerbation of both COPD and asthma by focusing on the clinical significance of associated respiratory virus infections. Furthermore, influenza A(H1N1)pdm09 virus have suddenly emerged in Mexico in the spring, 2009. The virus can cause influenza pandemy accompanying with pneumonia/wheezing. Obuchi et al. (2013) review essential reports with regard to asthma in patients infected with the virus, and they discuss the utility of influenza vaccines and antivirals. Although HPIV3 is an etiological agent for respiratory disorders such as pneumonia and asthma, there is no prophylactic human vaccine against the virus infection. In the 9th issue as original article, Senchi et al. (2013) present the development of an oligomannose-coated liposome (OML) nasal vaccine against HPIV3 in combination with an effective adjuvant Poly(I:C). They report that their newly-developed vaccine can successfully induce antigen-specific immunity with a small amount of antigen via the nasal route. These results highlight the utility of combining sophisticated systems in the development of a novel vaccine against HPIV3. In the final article, Matsunaga et al. (2014) present the development of monoclonal antibodies (MAbs) against hemagglutinin-neuraminidase (HN) of HPIV3. For synthesizing the antigen protein, they utilized the wheat germ cell-free system. This new cell-free system-based protocol for antigen production enabled to create the MAbs that can be applicable in various immune assays such as flowcytometry and immunoprecipitation analyses. The newly-developed MAbs could thus be a valuable tool for the study of HPIV3 infection as well as the several diagnostic tests of this virus.

In conclusion, we believe that these works are well summarized and informative to glimpse the field of virus-associated asthma/COPD, and may help understanding the basic and clinical aspects of the disease. We would be happy if this collection of papers will offer new stimuli and perspectives for not only researchers but also clinicians working around the exciting and emerging the e-book.

## Conflict of interest statement

The authors declare that the research was conducted in the absence of any commercial or financial relationships that could be construed as a potential conflict of interest.
